# Illustrations of Pulmonary Consumption, Its Anatomical Character, Causes, Symptoms and Treatment

**Published:** 1834

**Authors:** 


### REVIEWS AND BIBLIOGRAPHICAL NOTICES.

### Art. VI.—Pulmonary Consumption.

*Illustrations of Pulmonary Consumption, its Anatomical Character, Causes, Symptoms, and Treatment. With* 12 *Plates, drawn and colored from Nature.* By George Samuel Morton, M. D., Physician to the Philadelphia Alms-House Hospital, &c. pp. 183. Phil. 1834—Key and Biddle.

Of the various topics which divide the Profession in the present day, Morbid Anatomy is neither last nor least in the interest it excites. We are advocates for its study, but would not class ourselves with those *ultras*, who seem to regard it almost sufficient of itself, to qualify men for the practice of medicine. Were it the province of the profession to cure diseases after they had produced permanent alterations of structure, we should ascribe to it a higher grade of utility than we are now disposed to assign. All the world, however, is aware, that derangements of structure are, generally, irreparable; and that a knowledge of them, is only beneficial, by disclosing to us the seat and kind of diseased action, which had previously existed, and which was the proper object of practical medicine. As far then as it can thus instruct us, and no further, this study contributes to the advancement of therapeutic medicine; for the proper business of the physician is to subdue morbid action, and thus prevent morbid structure.

It will be conceded, that in acute maladies, the diseased actions which build up diseased structure, are, in most cases, those which are designated by the term *inflammation;* and that the varieties of morbid structure, found after death, generally result from the difference of morbid organization, in which lesion has taken place. If these propositions be admitted, it follows, that unless inflammation requires a different treatment, when seated in one tissue, from what is necessary when it invades another, it cannot be, in practice, of any great importance to know its exact locality, or what particular variety of disorganization it is effecting. Thus to borrow an illustration from the groupe of diseases which are the subjects of Dr. Morton's book, it does not signify very much, that the physician should know, whether a pectoral inflammation is seated in the pleura, the mucous membranes or the intervening cellular tissue, if, in all cases, blood-letting, antimonials, digitalis, and counter irritants are the remedies. But is it not true, that all inflammations, wherever located, and to whatever abnormal aspect the parts are tending, are to be met by the treatment which is called antiphlogistic? We think it is, and hence the first business of the physician, is to scrutinize the state of the functions, and decide that the condition of the system is inflammatory. Having done this, he proceeds to the use of antiphlogistics, and in all cases employs those which are generally the same. In this way we observe men, who know but little of morbid anatomy, successfully engaged as physicians, and see them cure diseases without being able, with any degree of certainty, to assign the *particular* tissue in which they are seated, or predict the morbid appearance after death, should their *modus medendi* prove ineffectual.

We would not, however, discourage the study of morbid anatomy, but only limit its importance to what seems to us sustainable ground; and hence we hail the appearance of Dr. Morton's book, as among the first contributions to this department of knowledge, which our countrymen have made; and hope it will be followed by many others equally faithful, especially in the graphical delineations, where the principal merit must necessarily lie.

Dr. Morton's chief object is to illustrate one form of pectoral disease—*pulmonary consumption;* but his first chapter presents a brief elementary view of the various diseases to which the lungs are liable, from which we shall extract the article MELANOSIS as a specimen.

"*Melanosis* is the name given by Laennec to masses of a black or blackish brown color, occasionally found in the lungs, especially in tuberculous persons. This matter, according to the later views in pathology, is merely an excess of secretion of the pigmentum nigrum, modified no doubt by disease; in fact it seems to bear much the same relation to the pigmentum nigrum that tuberculous matter does to the healthy albuminous secretions of the pulmonary tissue. Melanosis is mostly seen in amorphous masses, or irregular laminæ, as represented (Pl. III. fig. 2,) or in black spots in the cellular tissue immediately beneath the pleura. (Pl. XI. fig. 1.) It is sometimes encysted, and looks then not unlike a bronchial gland. Pathologists also speak of this secretion in the fluid form, a modification I have not witnessed. I am disposed to think that melanosis is less frequent in this country than in Europe. In the Parisian hospitals, Bayle met with it in about a twelfth part of his autopsies." p. 16.

The second chapter is devoted to the anatomy of tubercular matter; which, according to our Author, is found in two very different forms—concrete and gelatinous,—the former of which presents several varieties.

Of the concrete tubercle he recognizes, along with the latest and best writers, three kinds—1. Miliary tubercles, which are generally of a yellowish or greenish, gray color, varying in size from a mustard seed to a cherry stone, opake, or partially transparent, solitary or aggregated, and of various shapes. 2. Crude tubercles—the preceding variety tending to suppuration—color often a yellowish, white tint. 3. The gray semi-transparent matter, consisting of masses which are smooth, moist, and shining; when cut—apparently dense, but crumbling under the finger—the air cells of the part in which it is found entirely filled up or wanting.

"This variety, like the following one, manifestly results from an abundant secretion of the tuberculous matter through a large portion of parenchyma at the same time: masses very similar in appearance, however, result from the slower process of miliary accretions, especially when the latter, assuming an angular form, coalesce into a uniform mass, as may be inferred from Pl. II. fig. 2." p. 20.

Gelatinoid infiltration is sometimes harder, sometimes softer, than jelly; now and then semi-transparent and rose-colored, but oftener colorless, or grayish or olive. It fills up the interstices between the common tubercles, or pervades the cellular tissue in a homogeneous manner. It frequently presents the aspect of pure albumen; showing that the opinion of Cruvelhier, that tuberculous matter is secreted in a fluid state, is sometimes true.

"The gelatinoid infiltration is an example of tubercular matter secreted in the semi-fluid form; it is evidently a rapid secretion, and appears in some instances to be converted into crude tubercle by a process almost equally rapid, until the lung becomes a dense, and seemingly inorganic mass, without a trace of respiratory structure." p. 21.

Encysted tubercle is a rare occurrence. The cyst is fibrous or cartilaginous. In the dissection of a negro man, Dr. M. found the following example of this variety.

"*Right lung* firmly adherent above; the two upper lobes were loaded with tubercular matter in large masses, and mostly in the crude state: that in the superior lobe formed an ovoidal mass the size of a goose-egg, and was surrounded by a distinct, white, cartillaginous, cyst, about a line in thickness: the cyst touched the pleura laterally, but was easily separable from it, the pleura itself being much thickened and adherent at these places. The thin edge of the lung towards the mediastinum, was filled with tubercles, but the cyst formed a perfectly distinct boundary between them and its own contained mass, which was of a greyish yellow color, opaque, and mottled with darker points: its upper and posterior portions had already begun to suppurate, there being a number of small vomicæ that communicated freely with each other." p. 23.

Tuberculoid granulation (*granulationes miliaires* of Bayle.) Dr. M. with Andral, supposes to be the air cells of the lungs, dilated and filled up by inflammation. If so, they are totally distinct from the tubercle. After stating some facts touching this matter, our author adds—

"I am therefore inclined to believe that these substances hold the same relation to the gray induration of pneumonia, that tubercles bear to tubercular infiltration: and if they should be subjected to analysis (which I am not aware has ever been directed to these bodies in particular), they will probably prove to be composed of fibrine, and not, like tubercles, of albuminous matter." p. 25.

Dr. Morton refers to the indestructible character of the cellular tissues, and supposes that it surrounds and nourishes the tubercles, although its arteries and veins are perhaps generally obliterated early in the disease.

According to Sherard, 200 grains of unsoftened tubercular matter consisted of—

| | |
|---|---|
| Albuminous animal matter, | 98.15 |
| Muriate of soda, Phosphate of lime, Carbonate of lime, | 1.85 |
| Oxide of iron, a trace. | |
| | 100.00 p. 27. |

Tubercles, it is well known, are developed in all the tissues of the body except the horny, but are particularly common in the lungs. Their softening constitutes the essential anatomical character, of true pulmonary consumption. The term suppuration has been used to express this change, but suppuration commonly takes place in the surrounding parts, and is, perhaps, excited by the presence of the tubercle. When the contents of these abscesses make their way into the bronchial tubes, the tubercular and purulent sputa is expectorated, in the place of the mucus thrown off by the sympathizing mucous membrane. Cavities are now formed as a matter of course, and are of various sizes, according to the dimensions of the masses of tubercular matter. But few of these masses make their way to the bronchial vessels, and hence in cutting through the lungs, we in many instances, encounter small and confined collections. But a few days since, we saw the same thing in a tuberculous liver.

"The softened tubercular matter of abscess is opaque, of a caseous consistence and yellowish color; it at first more or less completely fills the cavities, and is evacuated, as the disease advances, by one or more bronchial tubes, which, leading from the abscess, have their lining membranes continuous with it. The matter of abscesses is sometimes hard, dry, deposited in successive layers to considerable thickness, and peels off like the coats of an onion.

We often find small abscesses completely filled with this semi-fluid caseous matter; but after they have been once emptied, it is observed chiefly in irregular masses on their parietes, while the function of the cavity consists in the elaboration of a fluid which has all the characters of pus.

This purulent matter varies in its color and consistence in the same way as in abscesses in other parts of the body; sometimes being straw-colored, thick, inodorous and viscid; again thin and serous, sanguineous, ichorous, fœtid, &c.

This secretion escapes from the cavity by one or more fistulous ducts, the remains of bronchial tubes, through which it is transmitted to the trachea and thence expectorated. But it occasionally happens, that when suppuration occurs in the midst of extensive tubercular masses in which no bronchial tubes remain, the abscess may continue for a length of time imperforate; finally, however, it meets with a bronchial ramification, and I have known the egress of pus to be so great and so sudden as at once to strangle the patient.

Physicians, since the time of Hippocrates, had given the name of *vomica* to abscesses of the lungs consequent to pneumonia. Laennec, however, from the supposed infrequency of phlegmonous abscesses in those organs, restricts the appellation to the cavities formed by the softening of tubercules." pp. 31—2.

Chapter third is devoted to the *pathology of phthisis*. It is well known to all who have examined those who died of this disease, that the upper part of the lungs is the chief seat of tubercles. Dr. M. ascribes this, *perhaps* correctly, to the compression which he supposes that part to suffer, more than any other, from the bony structure enclosing it.

Our author adopts the opinion of Andral, that the secretion of tubercular matter is a disordered state of the function of serous secretion, which exists in all the cells and areolæ of the body; and, with the author just quoted, as well as Bayle, Laennec and Louis, believes that tubercles are not the offspring of inflammation, as Broussais argues. We cannot entertain a doubt of this opinion, as in addition to the facts furnished by the distinguished pathological anatomists here enumerated, we have seen numerous *congenital* tubercles, in the liver, spleen, and intestinal parieties of a child, with the parts enclosing them perfectly sound.

On the destruction of large blood-vessels, in tubercular abscesses, our author has the following observations.

"The manner in which the blood-vessels are destroyed in tubercular disease, has never, that I am aware, been satisfactorily explained. The result of many observations directed to this point, leads me to the following conclusions: the cellular tissue constituting the outer coat of the artery, secretes its own tubercular matter, and preserves the form of the vessel until suppuration takes place. The middle coat of the vessel meanwhile preserves its red color; but between it and the internal coat a second layer of tubercular matter is observed, doubtless arising from a lamina of cellular tissue connecting those coats together. The inner coat, however, does not appear to change during the whole process of tubercular disease, but retains its pearly, diaphanous, and polished character. In order to trace this pathological condition of the blood-vessels, it is necessary to examine such of them as traverse a large tubercular mass, after the latter has become softened, but anterior to suppuration: if the vessels then be separated by cautious percolation with water, and a trunk be cut across, the several facts above mentioned will be rendered obvious. If, however, the examination be not made until after suppuration has taken place, the cellular tunic of the artery will be found to have granules or masses of tubercular matter adherent to it, not derived from the contents of the abscess, but from its own proper tissue: the portions intermediate between these granules are of a florid red color, and appertain to the denuded middle coat of the artery. This tuberculous degeneration of the blood-vessels is obviously derived from the vasa vasorum themselves." pp. 36—7.

Our author examines and rejects the theory of Sylvius, supported by Duncan and Broussais, that tubercles arise from an inflammation of the lymphatic glands, alledging that anatomy has not even shown the *existence* of such glands in the lungs, and finally concludes with Andral, that—

"The pathology of tubercular disease may, I think, be summed up in the following manner:

"1. Tubercles are an altered secretion of the albuminous halitus proper to the cellular tissue forming the parenchyma of organs.

"2. Inflammation is not necessary to their developement.

"3. The cellular tissue which envelopes and intersects tubercles, sooner or later takes on inflammation, and secretes pus; by which process the tubercular matter is eliminated, and an abscess is formed." p. 39.

The fourth chapter opens with an inquiry, whether we know of any physical signs, by which to determine that an individual has a tubercular diathesis, and whether it can be identified with the scrophulous predisposition, and the decision of both questions is in the negative; from which, relying on our own observations, we are not inclined to dissent. Still it cannot be doubted, that a great many persons have an hereditary tendency to this malady. Of the causes which originate this predisposition we know nothing certain; but a cold and variable climate, especially in the neighborhood of the sea, would appear to be one of the most efficient. It operates, we may presume, by disturbing the vaporous interstitial secretion, of which we have already spoken.

This brings us to consider the *exciting* causes of phthisis, the most profitable study which the whole subject presents.

Chronic catarrhal inflammation, leads to the production of tubercles, by disturbing the pulmonary circulation and secretion, and on the other hand tubercles, when they soften, not unfrequently excite bronchitis. Exposure of the surface of the body, inadequately promoted, to a cold and damp atmosphere, has, without exciting catarrh, promoted the formation, or the softening of tubercles. Mechanical irritation of the mucous membrane, may produce the same effect. In these cases, Dr. M. thinks the agitation of the lungs, may be the chief cause of the developement of a tubercular condition; but we may readily suppose, that the cellular tissue sympathises with the mucous. The measles sometimes excite the lungs to develope tubercles. Pneumonia appears to produce the same effect in two ways, by disturbing the cellular secretion of halitus, and by the permanent induration of a portion of the lungs, mechanically disturbing the circulation.

"There is another series of causes that acts primarily upon the apex of the lung, producing congestion, which is often rendered unequivocal by the appearance of hæmoptysis. Among these causes may be enumerated, excessive muscular exertion in lifting, running, boxing, rapidly ascending stairs, &c. Also loud and continued vociferation, whether in singing, laughing, shouting, or speaking; also the blowing of trumpets and other wind instruments.

Let me repeat, that in such efforts the respiration is temporarily suspended, and of course with it the arterialization of the blood, while the latter fluid rushes to the lungs in greater quantity and with greater velocity than ever. An apoplectic and congested state is the consequence, sometimes accompanied by hæmoptysis, at others by a rapid succession of alternating chills and heats, excessive languor, &c." pp. 44—5

Our author rejects hæmoptysis as a cause of tubercles, regarding them rather as a consequence. We recollect a case which occurred in our own practice, many years since, that might be cited in support of this conclusion. A gentleman, accustomed to write many hours at the desk every day, was seized with hæmoptysis, without having been at all aware of any disease in his lungs. He passed rapidly into phthisis, and died within a short time, of an inflammatory affection of the brain, apparently resulting from an obstruction to the blood in its passage through the lungs. On examination after death, his lungs were found tubercular throughout. But few of them had yet passed into a state of suppuration.

After referring to the pernicious effect of certain trades and occupations which keep the body in constrained and unnatural postures, our author adds—

"There is another source of consumption, the more to be deplored because it is sanctioned by the tyranny of fashion: I allude to the custom of *tight lacing*. It is well known that this practice, if commenced in younger life, and persisted in, greatly diminishes the lateral diameter of the chest, and necessarily cramps the lungs, deranges their functions, and disorganizes their structure. In those who are predisposed to consumption, I cannot imagine a more certain mode of inducing it." p. 45.

The debilitating passions, nostalgia, melancholy, and other affections of this kind, are all, in the opinion of the author, capable of promoting the formation or softening of tubercles.

Under the head of influence of age, he presents us with a tabular view of the epochs of life, at which 281 patients died in the Philadelphia Alms House, which we extract.

| | |
|---|---|
| "Under one year, | 3 |
| From one year to ten, | 1 |
| From ten to eighteen, | 4 |
| From eighteen to thirty-five, | 142 |
| From thirty-five to forty, | 51 |
| From forty to fifty, | 42 |
| From fifty to sixty, | 20 |
| From sixty to seventy, | 12 |
| From seventy to eighty, | 3 |
| From eighty to ninety, | 2 |
| From ninety to one hundred | 1" p. 48. |

To the question of the relative prevalence of phthisis in the two sexes, our author has not collected facts for an answer.

Chapter five is on the symptoms of consumption. We shall not undertake to analyze it, as they are set forth in all the books. The following case is not without interest.

"*Disappearance of cough nine days before death, and remarkable absence of the other symptoms of phthisis.*—John Brady, a mulatto hostler, aged twenty-nine, was admitted into the Philadelphia Alms-house hospital, in December, 1832, with a troublesome cough, extreme languor, and corresponding mental hebetude. He remained in the house all winter, and came under my care on the 1st of February, 1833. Complains of nothing but inordinate fatigue and weariness; assures me that he has never had hæmoptysis, pain, or night-sweats, nor have any of these symptoms been noticed since admission: coughs considerably, but does not appear to be distressed by it. Expectoration scanty. The stethoscope proves the respiration to be very dull in the upper half of the left lung.

February 6th. Cough and expectoration less than ever. Lethargic and almost unconscious.

February 8th. Cough and expectoration have ceased. Complete aphonia.

February 10th. Somewhat revived, but cannot speak. Retention of urine, which flowed freely through the catheter. Appears to suffer pain in the lumbar regions when moved. Involuntary fœcal evacuations.

February 14th. Pulse better; voice returned so as to be intelligible; urine and fœces under control of the will, but no cough; has, however, subsultus tendinum, facies hippocratica, and the same languor which has induced him to lie in one position, on his back, for nearly two weeks past.

February 17th. Died.

*Autopsy*, assisted by Drs. Mason and C. A. Porter.

The *right lung* was free, and charged with tubercles in all its lobes, but especially in the upper one; no vomicæ and no infiltration.

*Left lung* adherent throughout by slight membranous laminæ, and literally filled with tubercles in every stage of development, from minute granules to vomicæ, though the latter were mostly not larger than a small filbert. This lung could have subserved no part in the respiratory function for a considerable period before death.

*Heart* natural.

*Liver*, *kidneys*, and *bladder* healthy.

*Remarks* —This case is highly interesting, inasmuch as it proves to what extent tuberculous disorganization may proceed in the lungs, without producing the usual symptoms of phthisis." pp. 53—4.

The next two chapters are dedicated to the complications of phthisis with other maladies. Our author has met with four cases, in which it apparently gave rise to *fistula in ano.*

We have seen several. As it has occurred but seldom to Laennec and Andral, it would appear to be less frequent in Europe than the United States. Destruction and absorption of the bronchial tubes after the air cells, to which they lead, have been filled up, and dilitation of the bronchia, are consequences of phthisis. When the pulmonary abscesses approach to the pleura, inflammation of that tissue with consequent false membranes, may supervene. In some instances the ulceration has passed through the pleura pulmonum into the sac of the pleura, which becoming the receptacle of the tubercular and purulent matter, has been violently irritated. Gangrene of the lungs, occasionally supervenes on phthisis. A more remarkable complication, was a single case (too long to be extracted) in which a tuberculous abscess of the lung, communicated by a fistulous opening with an abscess on the back. After citing a case of phthisis, complicated with diseased liver and jaundice, our author has the following—

"Dr. Wilson Philip supposes that one-half the consumptions of Great Britain originate in hepatic derangements; yet he adduces but slender evidence of his opinion; nor do I hesitate to say, that in this country it is by no means corroborated. It is true that I have often observed functional derangement without being able to detect any organic lesion of the liver; but again, in a great number of cases, I have found that organ unimpaired.

"In truth, every practical pathologist must have had occasion to notice, that the liver is often greatly diseased without having given any prominent characters to the patient's symptoms; and again, there is often violent gastric irritation, with pain, nausea and vomiting, and other dyspeptic signs, without any evidence, from post mortem appearances, that the liver had any active part in the complication. I am satisfied that the hepatic affections are in most instances consecutive; for the adipose transformation (the most frequent of these affections) appears to be one of the derangements that follows, and is almost always dependent upon tubercular disease of the lung.

"It has even occurred to me, that persons who have disease of the liver, are less liable than others to tubercular consumption; and that where the lungs are involved by hepatic disease, is rather by the transmission of simple inflammatory action.

"The adipose transformation of the liver is not unfrequent: in these cases the surface preserves its smoothness, but is mostly of a dull yellow color speckled with red: within, it is either wholly or in part transmuted into a yellowish fatty substance, which in the early stage is irregularly mingled with the natural color of the liver, giving it a marbled or mottled appearance. I have chiefly noticed this change in persons of intemperate habits." pp. 95—6.

The cure of phthisis after the lungs have become cavernous from tubercular suppuration, could not be expected, and still such cases have occurred. Dr. M. has met with one which from the great infrequency of such encouraging instances, we shall transcribe, with his remarks:

"Case 28. *Complete cicatrization of the several tuberculous abscesses.*—A lady, aged thirty-two years, of strong constitution and good frame, but of a nervous temperament, with dark hair and brunette complexion, had been for some time under the care of Dr. Hodge, for an attack of severe nervous irritation, when, in the absence of that gentleman, I was requested to see her on the 6th of May, 1833. On my arrival I found her dying, and she survived but a few hours.

*Autopsy*, by Dr. Hodge and myself. There was no obvious emaciation.

The *ventricles of the brain* were nearly full of serum, but the organ itself was healthy, and no trace of tubercles was observed. The membranes, especially the pia mater, were highly inflamed and injected, the latter being charged with serosity, and having on its surface two or three small patches of inflammatory exudation.

The *thorax* was contracted in its antero-posterior diameter; and on removing the pectoral muscles, the five or six superior ribs were observed to be considerably depressed at their sternal extremities, where their cartilages joined them at a remarkable angle which protruded into the thorax. The *left lung* adhered at its apex, at which point the pleura was deeply contracted or puckered: within was observed a rounded white mass, about an inch in diameter, composed of granules of a cartilaginous firmness; this was obviously a cicatrized abscess; and in its centre were two or three crude tubercles. The remainder of the lung was perfectly healthy. The *right lung*, like the left, adhered at the apex, where the pleura was also deeply sunk and puckered: beneath one of these plications was the remains of an old, but very small abscess, half filled with granular matter like that in the other lung, excepting that it was of a darker color; the remainder of the abscess was in a suppurative state, and contained yellow pus. Close by were the evidences of a second cavity the size of a filbert, but perfectly filled and consolidated by white granular matter, precisely like that in the left lung. The remainder of the parenchyma was healthy, excepting only a small calcareous concretion towards the base of the lung.

The *liver*, *spleen*, *stomach*, *intestines* and other organs, were examined, and appeared to be unimpaired.

*Remarks.*—The unexpected morbid appearances of the lungs induced me to inquire into the previous history of the patient; when I

was informed by a near relative, that in early life she habituated herself to excessive tight lacing; but although she had never experienced any obvious ill effects from this practice, she had of latter years discontinued it, from a conviction of its injurious tendency.

It seems probable, therefore, all circumstances considered, that the lungs became tuberculous and cavernous, from the irritation of mechanical pressure; but on the latter being removed, the morbid secretions ceased, and the cavities became cicatrized and obliterated in the manner just mentioned. Can there be a doubt, that if this lady had persisted in the unnatural confinement of her respiratory organs, the tuberculous disease would have extended, the abscesses enlarged, and the disease become a fatal malady? The predisposition of phthisis being slight, it was suspended by the removal of the exciting mechanical cause; showing what important results physical education may produce on the human frame.*

Laennec seemed disposed to anticipate the cicatrization of abscesses in a much larger proportion of cases than experience is likely to realize. Besides, as in *Case* 27, one abscess with its fistulous canal may undergo this process at the same time that innumerable tubercles and numbers of abscesses remain in a state of active irritation: so that the cicatrization removes but a thousandth part of the disease, and affords no sensible relief to the patient.

"It may in general terms be assumed," says M. Laennec, "that when the sputa are yellow and opaque, the emaciation considerable, the hectic intense, these symptoms are less unfavorable when conjoined with manifest pectoriloquy, than when they exist without the latter phenomenon; because, in the first instance the symptoms may be attributed to the efforts of nature in softening and removing the tuberculous mass, and we may indulge the hope that they will cease when this process is completed, provided the remaining portions of the lung be in a healthy state. Whereas, in the second case, we may suppose the existence of a great number of tubercles, which will exhaust the patient before they can become softened and form ulcerated cavities."†

But the complete uncertainty of an accurate diagnosis in such cases must be obvious to every one; and although I have been able to record a solitary example of almost perfect cicatrization, and consequent spontaneous cure of what may be called a case of accidental phthisis, the instances are so rare that they must be regarded as extraordinary exceptions to a general law of nature." pp. 99—100—1.

Chapter 8th treats of the signs of phthisis, derived from percussion and auscultation. As we have gone fully into this subject, in our review of Laennec, in a former volume, we

* "Very straight lacing and straining for a fine shape, hath made many a fine girl spit blood, and ruined the lungs by preventing a full and free respiration." —Reid on Consumption, p. 199.

† L'Ausc. Med. t. i. p. 128.

shall be content with an extract on the signs afforded by the stethoscope.

"1. *Imperfect, or partial respiration.*—When tubercles are agglomerated, or when tubercular matter has infiltrated the parenchyma, the stethoscope of course yields a dull or diminished respiration; in fact, we often observe the respiration to be obsolete in some places, imperfect in others, and again full and sonorous. These phenomena, if repeatedly obtained from the superior lobe, leave scarcely a doubt of tubercular disease. But, as before stated, when tubercles are few, and dispersed in an otherwise sound lung, they cannot sensibly affect the vesicular respiration; nor can any physical signs with which we are acquainted, detect them under such circumstances.

"Unfortunately, however, this disease is not often stationary for any considerable period; and the changes which we have seen to characterize its second stage, are ascertained by the stethoscope with singular precision.

"2. *Cavernous respiration.*—When the tubercular matter has softened, and made its way into the bronchia, an open abscess is necessarily formed: the existence of this change is readily detected by the stethoscope, for the air can be distinctly heard to enter the cavity at every inspiration. When the cavity is large, and the bronchial tubes opening into it are small, the air enters the former with an audible puffing sound, which has received the name of *amphoric respiration.* Where the cavity contains considerable fluid, as frequently happens, in addition to the cavernous respiration we hear a gurgling sound, not unlike that produced by blowing gently into one end of a tube while the other is immersed in a phial of water. This is the *gargoulliement* of the French, and sometimes exists to such a degree, that there is no exaggeration in illustrating it by supposing a bellows to be used in place of the glass tube.

"3. *Pectoriloquy.*—This name has been given by Laennec, to a very singular phenomenon, which can only result from an abscess in the lung. Thus, if a cavity is situated near the surface of the lung, and at the same time communicates freely with the bronchia, every word the patient utters appears to traverse the stethoscope, and is conveyed to the ear of the operator as distinctly, in many instances, as the sound of the voice in conversation: such is *perfect pectoriloquy.* When, however, the cavity is small, or deep-seated, or has not free communication with the bronchial tubes, a modification of pectoriloquy is heard, called *imperfect,* because the sound of the voice appears to remain at the end of the stethoscope, not unlike the words of a person who speaks through a partition. Perfect pectoriloquy is an unequivocal proof of an abscess in the lung; though the existence of dense adhesions, the effusion of water into the pleura, and other causes, may prevent pectoriloquy from being heard, or at least render it obscure and doubtful, even where abscesses are evident from the other physical signs already noticed." pp. 116—17.

The next and last chapter, the longest in the book, is devoted to the treatment of phthisis. Anxious to know how this can be conducted with success, we labored with some impatience to reach it, but, as our fears suggested, we found nothing to gratify us. Whatever the profession can accomplish in the way of prevention, they certainly have not yet discovered how to cure tubercular phthisis; and we very much fear they never can. The occurrence, now and then, of a recovery by the cicatrization of the abscesses, and a cessation of the softening of the tubercles, both, the effect, apparently, of some spontaneous change in the constitution of the patient, holds out little encouragement to the physician as to the power of his recipes. Our author is fully aware of the actual state of the case, and, therefore, commences his chapter with instructions for the treatment of particular symptoms, in which we shall follow him; beginning with the—

"*Hemorrhagic symptoms.*—Pathological anatomy establishes three facts to guide us in the treatment of phthisis; 1st, That the disease primarily invades the apex of the lung: 2d, That it commences by congestion of the parenchyma: and, 3dly, That, in a majority of cases, the congestion is manifested by hæmoptysis. It follows, therefore, that the judicious treatment of the latter symptom is of the utmost importance to the patient, and consequently claims the scrupulous attention of the physician.

"In persons of general good health, in a first attack, the pulse being strong and the system feverish or excited, bleeding from the arm is indispensable; and the free and timely recourse to it may save the patient from the most hopeless consequences. Ten or twelve ounces of blood, taken rapidly from a large orifice, may divert the current of the circulation and relieve the pulmonary congestion. With this primary precaution may be associated some internal remedies, such as spirits of turpentine, elixir of vitriol, common salt, opium, sugar of lead, &c. Any one of these, repeated a few times at short intervals, will answer a good purpose. If by these means the hemorrhage is not speedily relieved, let two or three cups be placed on each infra-clavian region: but whether the cups be necessary or not, it is always desirable to establish a drain there, in the first place by a blister and subsequently by an issue, or tartar emetic plaster. Perfect rest, and a diet of gum water and farinaceous food, will generally restore the patient so much in the course of a week or ten days as to permit of removal to the country, which is of the utmost importance to convalescence; for what most promotes the general health, will most promote absorption of the congested blood,—without which process disorganization must rapidly follow.

"When, however, hæmoptysis takes place after a protracted and unequivocal manifestation of other phthisical symptoms, or when it recurs after an interval of two or three months, a totally different treatment is demanded. To bleed from the arm, or otherwise to reduce the strength of the patient, is in such circumstances inadmissible; for if by a cautious use of the stethoscope, aided by other signs, the lungs are found to be cavernous, I am convinced that active depletion, in any form, only tends to aggravate and hasten the disease. What good can be expected from reducing the strength of a person, who suffers a depletion from an abscess in the lungs? The most that we can do in this variety of hæmoptysis, is to check it by the internal use of the medicines above named, and the establishment of an issue below one or both clavicles. Tonic and alterative medicines, such as will be hereafter mentioned, are also extremely important.

"In the treatment of hæmoptysis, much often depends upon having the shoulders of the patient somewhat elevated, enjoining perfect rest, and prohibiting conversation." pp. 119—20—21.

We entirely concur with our author in the restrictions imposed on the practice of blood-letting in the last of these extracts; and are disposed, also, to unite with him in the following prohibition:—

"Once for all, I must denounce the practice of applying cold to the surface of the body to relieve pulmonary hemorrhage; it is contrary to every principle of pathology; and without materially checking the flow of blood, drives still greater quantities of it to the lungs, thus increasing the hemorrhagic congestion, and rendering the recurrence of the disorder more alarming than its onset." p. 122.

We should think, however, that the *inhalation of cold air*, in these cases, as proposed by Dr. Charles Drake, of New York, would be unobjectionable, and might be productive of good effects, if patients could be made to understand the difference between its application to the mucous and the dermoid surfaces, and, thereby, rendered willing to employ it.

*Catarrhal symptoms.*—Our author has found these removed by cups or leeches below the clavicles, gum water acidulated with lemon juice and containing a little morphia and tartar emetic. All very good, but bleeding from the arm and emetics are often more effective.

"The following prescription will be found a good one:

℞. Mucilaginis gummi arabici, oz. xvi.
Syrupi tolutani, ij.
Morphiæ sulphatis, gr. iss. vel ij.
Antimonii tartarisati, gr. ij. Misce et signa.

A table-spoonful to be taken every two hours. When from any cause the antimony is objectionable, it may be replaced by a dramch or two of sp. nitri dulcis." p. 123.

We have found in this and many other varieties of sub-acute pulmonary inflammation, the following *formula* of so much service, that although we have already asked the attention of our readers to it, in a former number, we feel constrained to repeat it here.

℞. Arab. Gum. Mucilag. ℥viij.

Digital. Purp. Tinct. Satur. ʒij.

Opii. Camph. Tinct. ʒij.

Antinc. Tart. gr. ij.

Misce.

A table-spoonful every 2, 4, or 6 hours. If much fever be present, the paregoric must be omitted. When the pulse has begun to feel the impress of the digitalis, it must be given more sparingly. Should the patient become costive, half an ounce of sulphate of magnesia, may be added.

Dr. M. very properly cautions his readers against the excessive use of *blisters* in this affection, as he has often seen (what has fallen under our own observation) an increase of fever from their great irritation.

*Hectic fever.*—Our author, near the beginning of phthisis, when the pulse is full and hard, recommends bleeding, but thinks, on the whole, that the lancet should be used sparingly. If the pulse become more frequent after bleeding, he avoids the repetition. The rule should have been stated more explicitly; as the *first* effect of bleeding, in all cases, is to render the pulse more frequent. Should it be more frequent in the next paroxysm, no further venesection is at that time admissible. Our author advises digitalis, mild aperients, acidulated mucilages, and those ancient and venerable panaceas, the neutral mixture, and sweet spirit of nitre. At an early period the patient should resort to carriage riding. To abate the *colliquative sweats*, the surface of the body should be sponged with a solution of alum in proof spirits, in the proportion of an ounce of the former, to a pint of the latter, with the internal use of elixir vitriol in sage tea.

*Pleuritic symptoms* are to be met by local bleeding, a blister, and subsequently a poultice of bran and flax seed, as advised by Broussais.

*The gastric affections* attendant on phthisis, are relieved by leeches, or an embrocation of equal parts of spirits of turpentine, sweet oil and laudanum.

"*Diarrhœa*, in whatever stage of phthisis it may occur, is most readily subdued by injections of morphia dissolved in gum water, flaxseed infusion, or some other bland mucilage. It is well, at the same time, to give occasional table-spoonful doses of simple camphor water, which conduces to the same end, and has also a slightly anodyne effect.

"I have also employed with signal benefit a mixture of camphor water, laudanum and nitric acid: and where other means have failed, very small doses of calomel, combined with ipecacuanha and opium, will be found effectual. The bark of the common dog-wood (cornus florida) made into a pretty strong infusion, constitutes a valuable auxiliary.

"I have also sometimes used the Moorish apozem mentioned by Dr. Good from M. Orban, consisting of a grain of alum with a grain and a half of sulphate of iron, morning and evening, which is a most effectual check on diarrhœa." pp. 126—27.

*For constipation*, bran bread with cream will generally be sufficient.

Our author's method of treating particular symptoms, is followed by a series of observations on various remedial measures.

*Digitalis*.—We concur with him as to the benefits, far short of a cure however, which this powerful medicine may be made to produce; but in the advanced stages of phthisis, it must be given with great caution, and always in connexion with some preparation of opium.

"*Iodine and its preparations*.—Every physician is now familiar with the internal use of iodine in phthisis; and having myself used it very extensively, I am able to express an unequivocal opinion respecting it. In a large number of instances it has appeared, especially in incipient consumption, to arrest or suspend the tubercular secretion, and with it the hectic, marasmus, cough, dyspnœa, and other urgent symptoms. In administering it I have adopted the common formula, combining it with the hydriodate of potash; but I am cautious to discontinue it whenever it is followed by sick stomach, vertigo, or any of those symptoms usually called *nervous:* for, notwithstanding the assertion of Dr. Coindet, of Geneva, that he has never seen injury result from its use, several instances have occurred to me, in which the persistence in it would have certainly terminated in very unpleasant consequences.

"There are again some constitutions in which it does not appear to produce any obvious effects, either for better or worse; but in a majority of cases, even in the second stage of phthisis, I have been much gratified with the results. Thus it often relieves the dyspnœa, improves the complexion, and restores the appetite, even when the advanced progress of the disease precludes all hope of recovery. A lady has assured me that whenever her cough, dyspnœa and febrile sensations warn her of a fresh accession of disease, the use of the iodine at once dispels the symptoms, and restores her to usual health. In another marked case, that of a middle aged man, one of whose lungs has been in a state of abscess for eight months past, I have repeatedly rescued him from alarming relapses by the iodine mixture alone.

"In some instances it has so obviously improved the nutritive function, that patients have increased in flesh by its use, and at the same time recovered, in a considerable degree, a naturally florid complexion.

"I prescribe iodine at every period of phthisis, avoiding it only when there is much febrile excitement, or when it produces the objectionable consequences already mentioned: but its use should be governed by the adage, that 'there is nothing in our art that does good but may also do harm.'" pp. 130—31.

Of the *Prussic acid* Dr. M. says nothing new. He has used the *uva ursi*, as recommended by Dr. Bourne, of Oxford, Eng., with decided advantage; especially in the chronic catarrhs of aged people which simulate consumption. The *compound extract of sarsaparilla*, prepared in this city after the formula of Carpenter, he regards as a very valuable "alterative," having in some cases seen it "produce a renovation of health truly surprising." When consumption is obviously in connexion with a scrophulous diathesis, he gives the extract combined with hydriodate of iron. *Tonics* he thinks often admissible, and prefers the wild cherry tree bark, (*prunus virginiana*) and the serpentaria to all others. We have supposed that the former owes part of its efficacy, to the Prussic acid which it contains. *Narcotics* he would avoid, except when indispensable to suspend the cough and procure sleep.

"*Fumigation and inhalation.*—It is by these means only that substances can be applied directly to a diseased lung; and when we recollect that vapours are, by this process, freely received into all the air-passages and cells, and even into the abscesses, with which the bronchia almost invariably communicate, how important is it that we should avail ourselves of this adjuvant in the treatment of consumption!

"But the careless manner in which inhalation is usually regulated, has brought it greatly into disuse; and in truth, I have, from this cause, sometimes seen it do more injury than good.

"The common plan of burning tar, resin, myrrh, and other substances in the chambers of the sick, I have found to add to the distress of the patient, owing to the disengagement of an empyreumatic smoke.

"After a fair trial with various substances, there is no one which I have prescribed in this form with equal success to tar in combination with subcarbonate of potash, in the manner recommended by Sir Alexander Crichton.

"Thus an ounce of potash is added to every pound of tar, in order that the latter may be deprived of its pyroligneous acid. The two ingredients being well mixed, should be first boiled for a few minutes in the open air, in order to disengage any impurities, and then be kept at a simmer in the room of the patient. This is readily effected by putting the composition in an iron vessel, and placing the latter over a spirit lamp, or some analogous contrivance.

"In this way not only a chamber, but an entire house, is speedily pervaded with a most agreeable vapour, which, although it may excite some disposition to *cough*, both in healthy and sick persons, very soon, in a great majority of cases, allays this symptom, and with it a great proportion of the patient's distress. In truth, I have seen it act like a charm. The very first case in which I employed it, was that of a lady who had recently lost both a brother and a sister by consumption, who herself had a lung disorganized by tubercular disease, and a constitution that had already suffered greatly from this cause. From the day that she commenced the tar inhalation her cough almost entirely disappeared, and all her other symptoms became, and still continue, greatly alleviated, excepting the pleuritic pain between the shoulders. Unpromising as this case was for a first experiment, the result was so pleasing that I gladly extended the same means to other patients; and I can most strongly recommend it in tubercular consumption, as a palliative of its most harassing symptoms, and in the catarrhal and pneumonic forms of phthisis as a cure. But I agree with the author just quoted, that in any case where the skin is hot and dry, and the expectoration scanty, the tar vapour can scarcely be of service; and I am also free to acknowledge, that instances have occurred to me wherein the preceding symptoms were absent, and yet the vapour appeared to irritate, rather than tranquillize, the pulmonary organs. These exceptions, however, have been very few, and such as necessarily occur in any plan of treatment. In chronic catarrh, especially when attended with ulceration of the mucous membrane, (catarrhal consumption of the systems,) I know of no plan of treatment that can vie with this:

the same remark will apply to those morbid conditions left by pneumonia and pleurisy, especially when accompanied with purulent expectoration and dyspnœa." pp. 134—35.

After speaking of *setons*, *issues*, and the *tartar ointment* with approbation, our author observes—

"The time to interpose this class of remedies with effect, is in the onset of consumption, when a dry cough, burning of the hands and feet, pain and dispnœa, warn the patient of impending danger; and there is this additional advantage to be derived from issues, that they do not interfere with the employment of active exercise, or long journies. I have had patients who have kept their issues open while travelling hundreds of miles over rough roads, without experiencing from them any appreciable inconvenience." p. 138.

We have not room to say any thing on the subject of *diet and clothing*, and the observations of the author present nothing particularly new.

His remarks on *exercise* are substantially the same with those of Sydenham, reiterated by Rush, and lately insisted upon by Dr. Parrish, in a paper which we have already analyzed.

To the important subject of change of climate, our author has devoted a number of pages, but as we shall prepare a distinct article on that subject, for our next number, we shall not enter upon it now.

Dr. Morton's concluding observations, are chiefly on the possibility of a radical cure in tubercular consumption, which he thinks may sometimes take place from the *absorption* of the tuberculous matter. The following extract will present his views:

"It is altogether probable that tubercles are sometimes absorbed; nor is it improbable, although not wholly susceptible of proof, that many of the hectic fevers which are cured by change of climate, and other means, may have been cases of incipient tuberculous disease that have been removed by the timely interposition of art.

"Even where tubercular matter is not absolutely thus removed from the lungs, its progress may be, and often is, arrested and kept at bay, until the system recovers its wonted vigor, and the morbid matter ceases to irritate. I have now in my occasional charge, persons whose lungs have certainly been tuberculous for nine or ten years, and whose health is not sensibly more impaired than it was at the onset. I lately attended a man sixty-six years of age, who died of tuberculous abscesses in both lungs: he assured me that he had experienced marked consumptive symptoms thirty years previous, and suffered particularly from profuse hæmoptysis, which, however, never recurred. His symptoms eventually assumed the acute character, and he was not confined to his bed more than three months.

"A forcible illustration of the arrest or absorption of tubercular matter, has been mentioned to me by a distinguished member of the profession, who, however, at the moment of communicating the facts, was unable to refer me to the work in which they are published: they are as follow:—While Louis Bonaparte was king of Holland, a division of the French army, five thousand strong, raised in the southern provinces of France, was marched into Holland and quartered there. Tubercular consumption, in an aggravated and acute form, soon made its appearance among these young men, and so great were its ravages that hundreds were soon destroyed by it. In this state of things, the physician in chief applied to the government to save the remains of the army, by sending them back to France; which was done accordingly, excepting in the case of those who were already too ill to remove from the hospitals. Among those who were able to return, were many already in the incipient stage of the disease, and whose symptoms were becoming daily worse; yet even these became convalescent on reaching the south of France, and no deaths by consumption occurred after the troops left Holland.

"These facts show, in a remarkable manner, the effects of climate in producing phthisis, as well as in arresting, if not eradicating it." pp. 161—62—63.

We have thus given a pretty full analysis of the work before us, especially its therapeutic department, presuming, in reference to the treatment, that Dr. Morton's industrious researches, bring down the discoveries of the profession to the present time. Our readers will unite with us in the regret, that he has not enabled us to present them with any remedies of greater novelty and promise, than those which have been enumerated.

We shall only add, that the plates attached to the work are well executed and instructive, and conclude by expressing the hope, that Dr. Morton will keep his attention directed upon this important subject.

